# Supplementary material for: Velocity Spectrum Imaging Using Velocity Encoding Preparation Pulses
Source: Magn Reson Med. 2025 Dec 10;95(5):2568–79. doi: 10.1002/mrm.70218 (PMC12937115; doi:10.1002/mrm.70218)
Supplement: Supplementary file 1 — Figure S1: (A) Orthogonal sections of the water fraction at three different velocities (columns) along the main Cartesian axes (rows) for participant 1. The color scale indicates the fraction of spins moving at a specific velocity for each voxel. As indicated in the main text, the spatial global mean at each velocity was computed and used to regress out drift effects from the spectrum at each voxel. (B) Single slice views of same velocity spectrum along each axis (rows). Again, the color scale indicates the fraction of spins moving at a specific velocity for each voxel. For clarity, we only display every 5th (of 61) velocity bin between +/−2.175 cm/s, including the 0 cm/s velocity bin. Figure S2: (A) Orthogonal sections of the water fraction at three different velocities (columns) along the main Cartesian axes (rows) for participant 2. The color scale indicates the fraction of spins moving at a specific velocity for each voxel. As indicated in the main text, the spatial global mean at each velocity was computed and used to regress out drift effects from the spectrum at each voxel. (B) Single slice views of same velocity spectrum along each axis (rows). Again, the color scale indicates the fraction of spins moving at a specific velocity for each voxel. For clarity, we only display every 5th (of 61) velocity bin between +/−2.175 cm/s, including the 0 cm/s velocity bin. Figure S3: (A) Orthogonal sections of the water fraction at three different velocities (columns) along the main Cartesian axes (rows) for participant 3. The color scale indicates the fraction of spins moving at a specific velocity for each voxel. As indicated in the main text, the spatial global mean at each velocity was computed and used to regress out drift effects from the spectrum at each voxel. (B) Single slice views of same velocity spectrum along each axis (rows). Again, the color scale indicates the fraction of spins moving at a specific velocity for each voxel. For clarity, we only display ever [file MRM-95-2568-s001.docx]

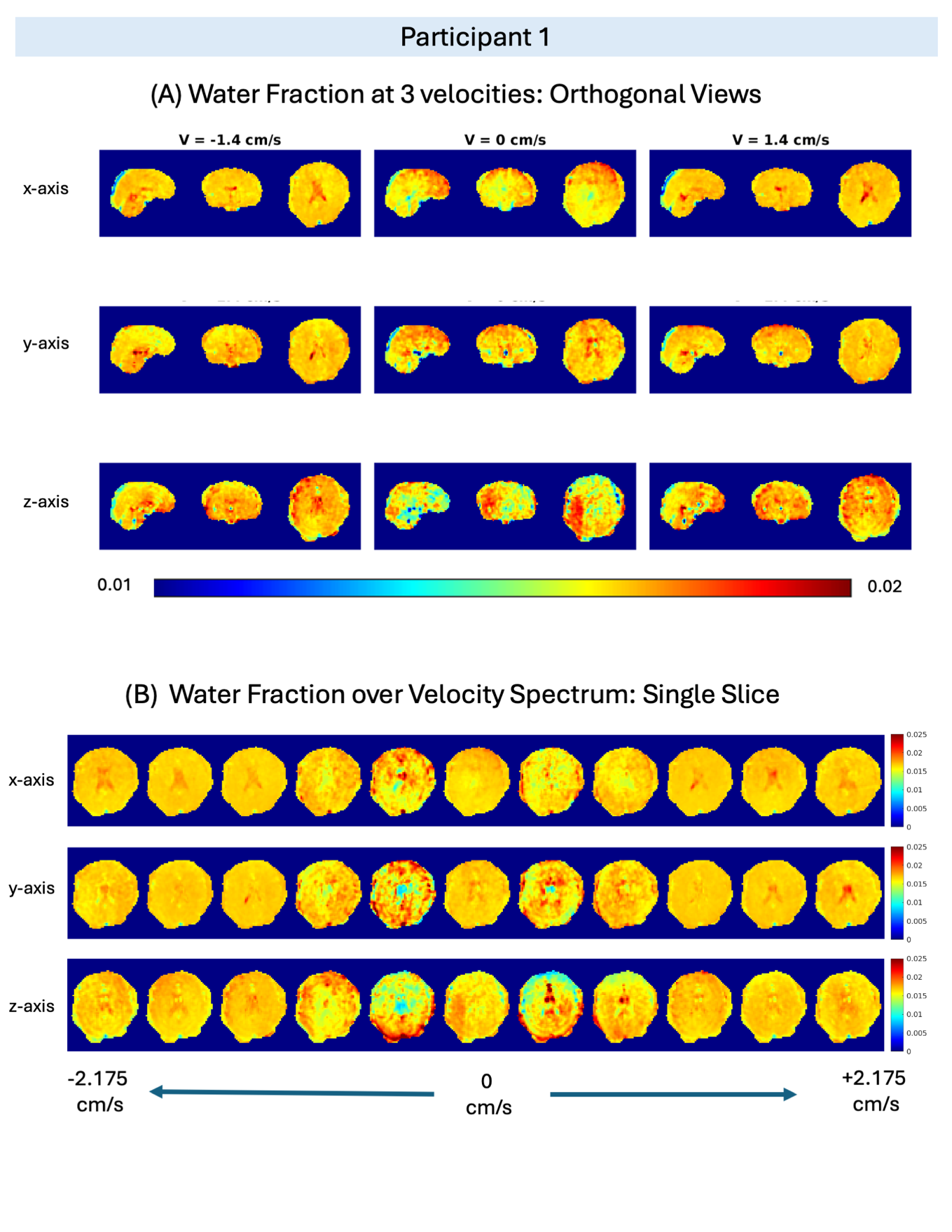
Supplemental Figure S1. (A) Orthogonal sections of the water fraction at three different velocities (columns) along the main Cartesian axes (rows) for participant 1. The color scale indicates the fraction of spins moving at a specific velocity for each voxel. As indicated in the main text, the spatial global mean at each velocity was computed and used to regress out drift effects from the spectrum at each voxel.

(B) Single slice views of same velocity spectrum along each axis (rows). Again, the color scale indicates the fraction of spins moving at a specific velocity for each voxel. For clarity, we only display every 5^th^ (of 61) velocity bin between +/-2.175 cm/s, including the 0 cm/s velocity bin.


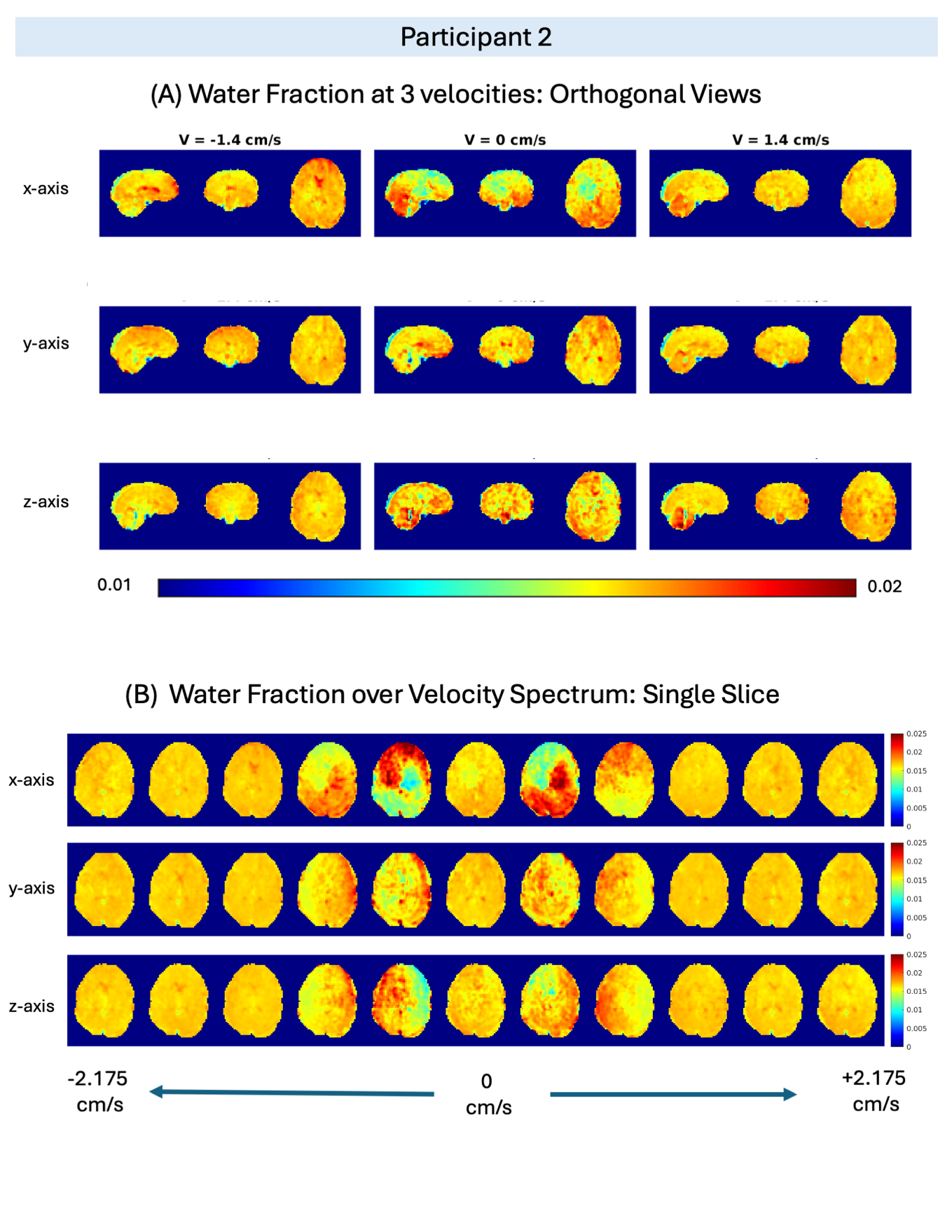
Supplemental Figure S2. (A) Orthogonal sections of the water fraction at three different velocities (columns) along the main Cartesian axes (rows) for participant 2. The color scale indicates the fraction of spins moving at a specific velocity for each voxel. As indicated in the main text, the spatial global mean at each velocity was computed and used to regress out drift effects from the spectrum at each voxel.

(B) Single slice views of same velocity spectrum along each axis (rows). Again, the color scale indicates the fraction of spins moving at a specific velocity for each voxel. For clarity, we only display every 5^th^ (of 61) velocity bin between +/-2.175 cm/s, including the 0 cm/s velocity bin.


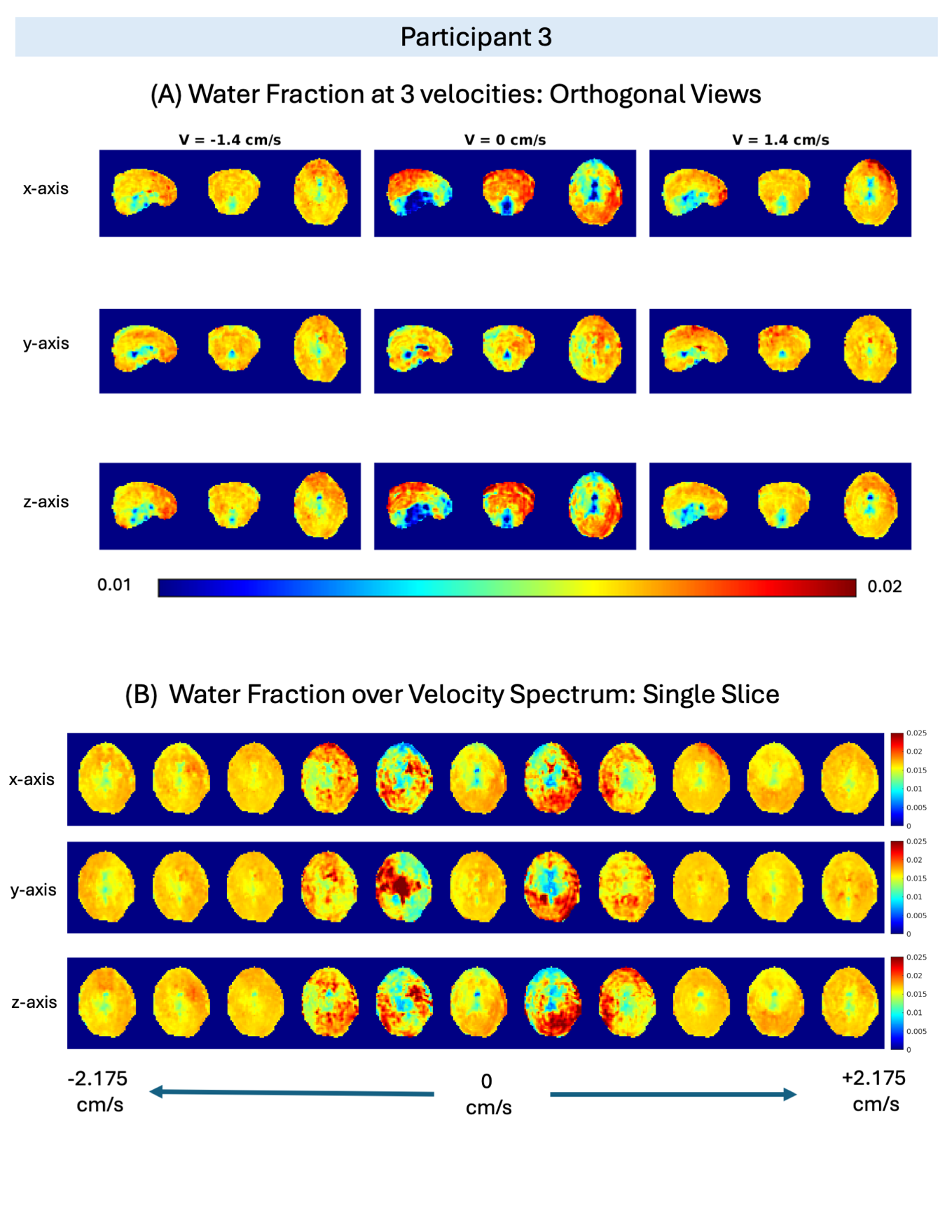


Supplemental Figure S3. (A) Orthogonal sections of the water fraction at three different velocities (columns) along the main Cartesian axes (rows) for participant 3. The color scale indicates the fraction of spins moving at a specific velocity for each voxel. As indicated in the main text, the spatial global mean at each velocity was computed and used to regress out drift effects from the spectrum at each voxel.

(B) Single slice views of same velocity spectrum along each axis (rows). Again, the color scale indicates the fraction of spins moving at a specific velocity for each voxel. For clarity, we only display every 5^th^ (of 61) velocity bin between +/-2.175 cm/s, including the 0 cm/s velocity bin.


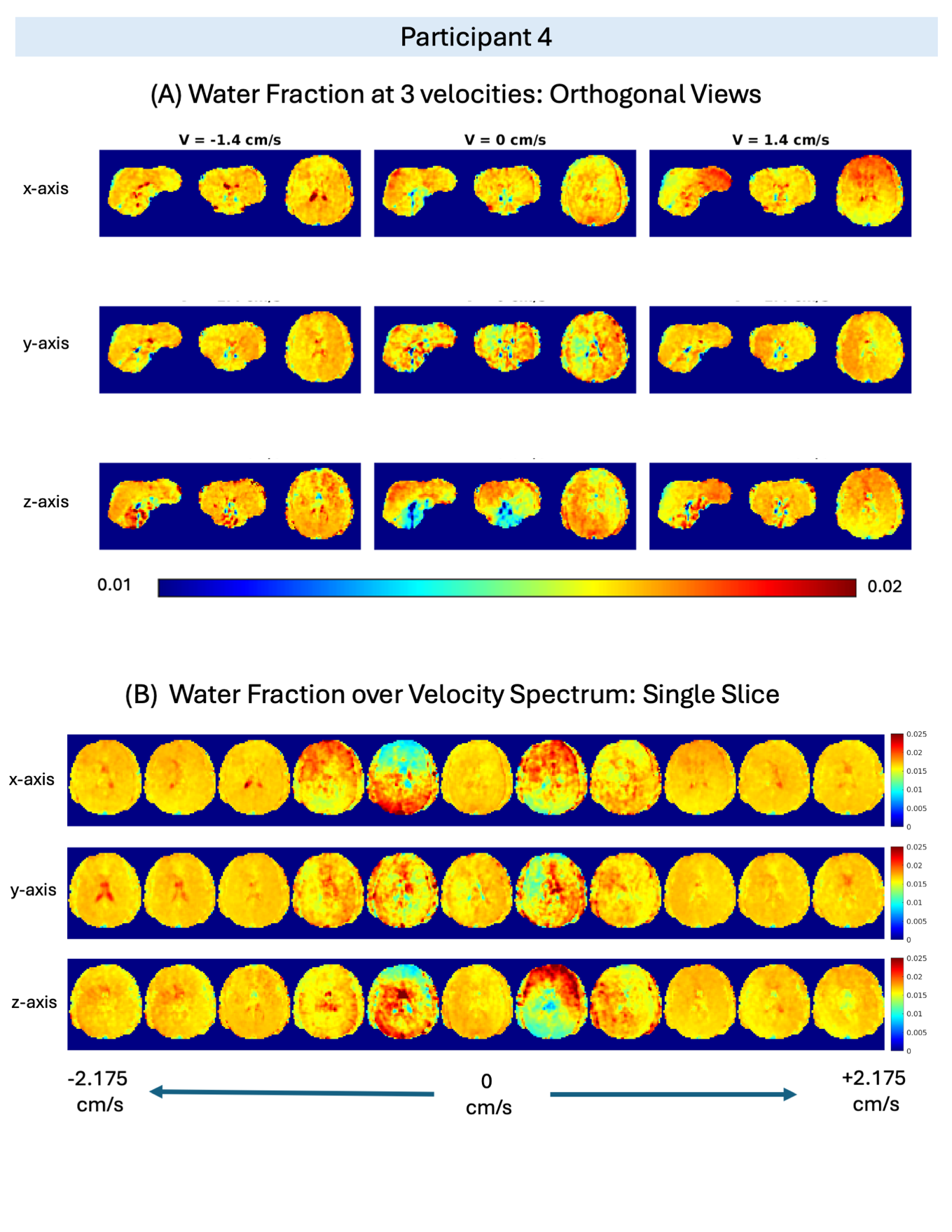


Supplemental Figure S4. (A) Orthogonal sections of the water fraction at three different velocities (columns) along the main Cartesian axes (rows) for participant 4. The color scale indicates the fraction of spins moving at a specific velocity for each voxel. As indicated in the main text, the spatial global mean at each velocity was computed and used to regress out drift effects from the spectrum at each voxel.

(B) Single slice views of same velocity spectrum along each axis (rows). Again, the color scale indicates the fraction of spins moving at a specific velocity for each voxel. For clarity, we only display every 5^th^ (of 61) velocity bin between +/-2.175 cm/s, including the 0 cm/s velocity bin.


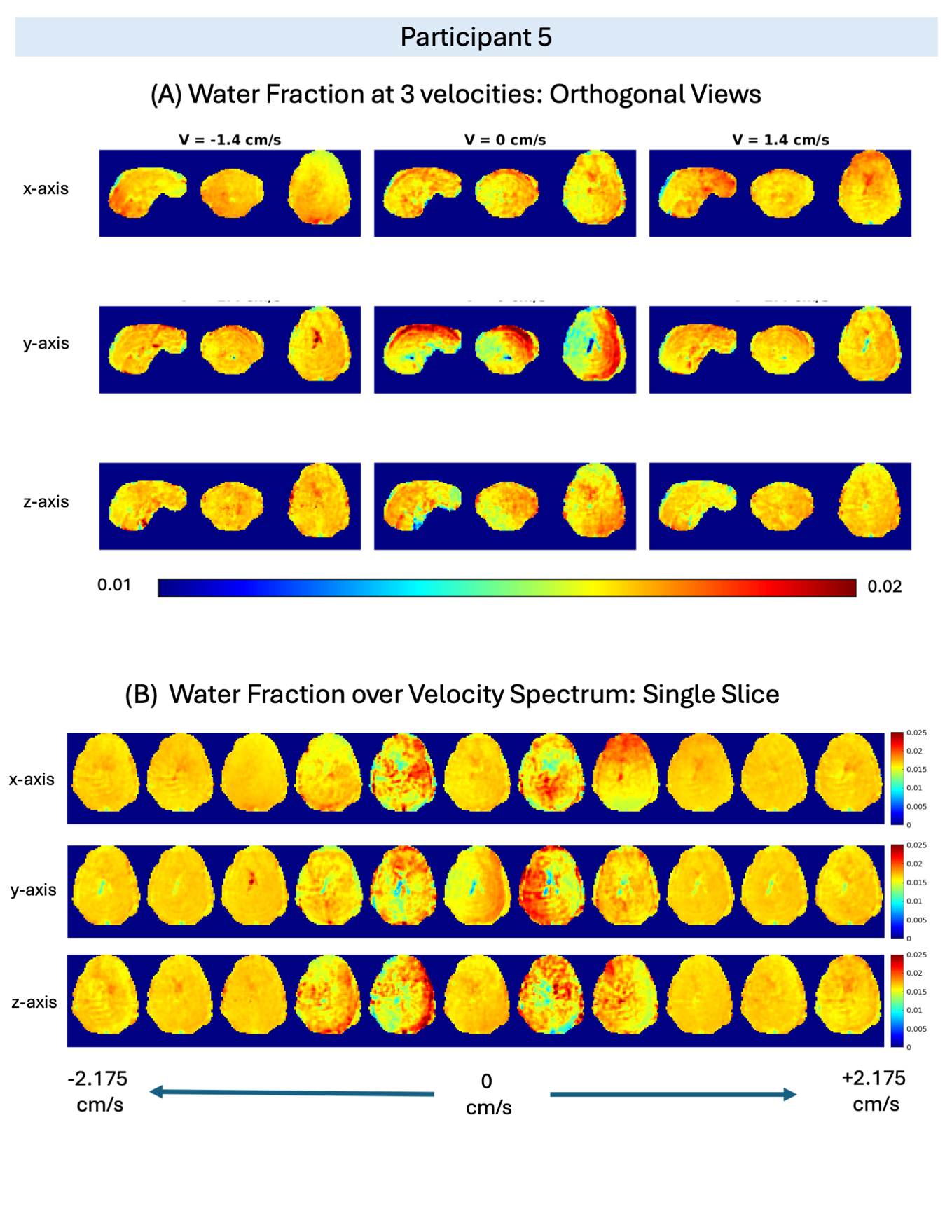
Supplemental Figure S5. (A) Orthogonal sections of the water fraction at three different velocities (columns) along the main Cartesian axes (rows) for participant 5. The color scale indicates the fraction of spins moving at a specific velocity for each voxel. As indicated in the main text, the spatial global mean at each velocity was computed and used to regress out drift effects from the spectrum at each voxel.

(B) Single slice views of same velocity spectrum along each axis (rows). Again, the color scale indicates the fraction of spins moving at a specific velocity for each voxel. For clarity, we only display every 5^th^ (of 61) velocity bin between +/-2.175 cm/s, including the 0 cm/s velocity bin.
